# Epithelial-to-Mesenchymal Plasticity in Circulating Tumor Cell Lines Sequentially Derived from a Patient with Colorectal Cancer

**DOI:** 10.3390/cancers13215408

**Published:** 2021-10-28

**Authors:** Pelin Balcik-Ercin, Laure Cayrefourcq, Rama Soundararajan, Sendurai A. Mani, Catherine Alix-Panabières

**Affiliations:** 1 Department of Molecular Biology and Genetics, Gebze Technical University, Kocaeli 41400, Turkey; pbalcik@gtu.edu.tr; 2 Laboratory of Rare Human Circulating Cells, University Medical Center of Montpellier, 34093 Montpellier, France; l-cayrefourcq@chu-montpellier.fr; 3 CREEC/CANECEV, MIVEGEC (CREES), Université de Montpellier, CNRS, IRD, 34943 Montpellier, France; 4 Department of Translational Molecular Pathology, University of Texas MD Anderson Cancer Center, Houston, TX 77030, USA; RSoundararajan@mdanderson.org (R.S.); mani@mdanderson.org (S.A.M.)

**Keywords:** circulating tumor cells, epithelial-to-mesenchymal transition, mesenchymal-to-epithelial transition, metastasis-competent cells

## Abstract

**Simple Summary:**

Metastasis is a complex dynamic multistep process; however, our knowledge is still limited. Very few circulating tumor cells (CTCs) are metastatic precursor cells and represent the intermediate stage of metastasis. Epithelial–mesenchymal plasticity (EMP) has crucial roles in tissue development and homeostasis, and also in metastasis formation. In this study, we explored the EMP phenotype of a unique series of CTC lines, obtained from a patient with colon cancer during the disease course and treatment, by detecting markers involved in the epithelial–mesenchymal and mesenchymal–epithelial (MET) transitions. This study shows that these colon CTC lines have acquired only few mesenchymal features to migrate and intravasate, whereas an increase of MET-related markers was observed, suggesting that metastasis-competent CTCs need to revert quickly to the epithelial phenotype to reinitiate a tumor at a distant site.

**Abstract:**

Metastasis is a complicated and only partially understood multi-step process of cancer progression. A subset of cancer cells that can leave the primary tumor, intravasate, and circulate to reach distant organs are called circulating tumor cells (CTCs). Multiple lines of evidence suggest that in metastatic cancer cells, epithelial and mesenchymal markers are co-expressed to facilitate the cells’ ability to go back and forth between cellular states. This feature is called epithelial-to-mesenchymal plasticity (EMP). CTCs represent a unique source to understand the EMP features in metastatic cascade biology. Our group previously established and characterized nine serial CTC lines from a patient with metastatic colon cancer. Here, we assessed the expression of markers involved in epithelial–mesenchymal (EMT) and mesenchymal–epithelial (MET) transition in these unique CTC lines, to define their EMP profile. We found that the oncogenes MYC and ezrin were expressed by all CTC lines, but not SIX1, one of their common regulators (also an EMT inducer). Moreover, the MET activator GRHL2 and its putative targets were strongly expressed in all CTC lines, revealing their plasticity in favor of an increased MET state that promotes metastasis formation.

## 1. Introduction

Cancer is the second leading cause of death in the world, and metastasis is the major cause of cancer-related death [1,2]. Despite the significant progress in cancer research, diagnosis, and treatment, the molecular mechanisms underpinning cancer cell dissemination are poorly understood [3]. Metastasis is a dynamic and complex process in which some cancer cells dissociate from the primary tumor, gain migratory potential, and enter the blood circulation to reach and colonize specific distant organs, where they may find favorable conditions to grow and form a secondary tumor [4]. These rare cells are called circulating tumor cells (CTCs), and might originate from primary or metastatic tumors. CTCs reflect the tumor burden, and harbor similar mutations as the primary tumor but also mutations specific of these more aggressive cells. They also highlight the presence of minimal residual disease, and can be used to detect a metastatic recurrence [5]. These cells are a unique and crucial source of information to understand the metastatic process [6,7,8]. Although various biological and molecular tumor markers and metastasis cascade mechanisms have been revealed, our understanding of this process is still limited. To intravasate and join the bloodstream, tumor cells must lose epithelial polarity and gain migratory capacity to pass through the basement membrane. This process is called epithelial-to-mesenchymal transition (EMT) [9]. Conversely, for the successful development of a metastasis in distant organs, cancer cells must revert to their original phenotype by re-expressing epithelial proteins and losing mesenchymal features (i.e., mesenchymal-to-epithelial transition, MET). Although EMT and MET have different effects on cancer dissemination, EMT- and MET-specific transcription factors play a role in similar cellular processes, such as embryonic development and cell differentiation [10]. During embryonic development, cells display epithelial–mesenchymal plasticity (EMP), with a dynamic flux between EMT and MET markers [11]. For the purposes of this manuscript, EMP is defined as the co-existence of epithelial and mesenchymal markers in CTCs to allow moving from EMT to MET.

ZEB1/2, SNAIL1/2, TWIST1/2, FOXC2, and Sine oculis homeobox 1 (SIX1) are transcription factors that induce EMT. Moreover, some cytokines (e.g., TGFβ and TNFα) induce these EMT markers in various tumors [12,13]. GRHL2, ELF3, ELF5, and OVOL1/2 are transcription factors that induce MET [14]. MET can also be induced by some transcription factors that suppress EMT activator pathways. Recent in silico, in vitro, and in vivo studies showed that EMT/MET features can be observed in different cancer cell line subtypes as well as in primary tumors. CTCs are an important class of cells exhibiting EMP [14,15,16,17]. Five years ago, our group established the first colon CTC line from a patient with metastatic colorectal cancer before treatment initiation, and then eight other CTC lines during disease progression and treatment (Figure S1) [18,19]. These nine CTC lines from the same patient present an intermediate phenotype with different expression levels of epithelial and mesenchymal markers. A partial EMT phenotype has been observed in CTCs by different groups [9,20,21,22]; however, it is quite difficult to thoroughly analyze this plasticity in rare single CTCs.

Therefore, we decided to characterize the EMP phenotype of these nine colon CTC lines because (i) they are metastasis-competent CTCs [18,19], (ii) they can be harvested in large numbers due to their efficient growth in culture, and (iii) they are from the same patient, thus allowing a longitudinal study of EMP features during tumor progression. Here, we analyzed the expression profile of transcription factors involved in EMP in the different CTC lines generated during the patient’s treatment (treatment-induced clonal selection process). We then compared the results obtained in the nine colon CTC lines with those of well-characterized cell lines derived from primary (HT-29) and metastatic (SW620) colon cancer.

## 2. Materials and Methods

### 2.1. Cell Culture

The colorectal cell lines HT-29 (ATCC HTB-38) and SW620 (ATCC CCL-227) were used as controls. HT-29 cells were maintained in Dulbecco’s Modified Eagle’s medium (DMEM) supplemented with 10% fetal calf serum (FCS). SW620 cells were cultured in RPMI 1640 medium with L-glutamine and 10% FCS. The colon CTC lines were cultured in CTC culture medium as previously described [18,19]. All cell lines were maintained at 37 °C in 5% CO_2_.

### 2.2. TGF-β Stimulation

For TGF-β stimulation, all CTC lines and SW620 cells were cultured in 24-well plates (Falcon, Corning, NY, USA) and incubated with 10 ng/mL of human recombinant TGF-β1 protein (Ref: PHG9214, Gibco by Life Technologies, Carlsbad, CA, USA) in complete culture medium for six days. The culture medium was replaced every two days with fresh medium containing TGF-β1, and cells were passaged just before reaching 70% of confluence.

### 2.3. RNA Isolation and Microarray Analysis

Total RNA from each sample was extracted using the RNeasy Mini Kit (74106-Qiagen), as recommended by the manufacturer. The RNA concentration of each sample was measured using a NanoDrop One spectrophotometer (ThermoFisher, Waltham, MA, USA), and a solution of 100 ng/µL was prepared for microarray analysis with the Affymetrix GeneChip. RNA quality was analyzed with a Bioanalyzer 2100 (Agilent, Santa Clara, CA, USA). Total RNA (200 ng) was used to prepare cRNA using the Affymetrix 3′ IVT express protocol (ref. 901229). cRNA was amplified by in vitro transcription. Amplified RNA (aRNA) was quantified with a NanoDrop ND-1000 spectrophotometer. After fragmentation, 12 µg of labeled antisense aRNA was hybridized to HGU133 plus 2.0 GeneChip arrays (Affymetrix^®^, Santa Clara, CA, USA). Microarray data were obtained and analyzed using the Transcriptome Analysis Console (TAC) software.

### 2.4. RT-qPCR

Complementary DNA (cDNA) was obtained by reverse transcription (RT) with the SuperScript III First-Strand Synthesis Super Mix kit (18080, Invitrogen, Waltham, MA, USA) according to the manufacturer’s instructions: 1 µL of each RT product was added to 9 µL of mix containing the 2 primers (6 µM each) and Brilliant III Ultra-Fast SYBR Green Master Mix (600882, Agilent Tech., Santa Clara, CA, USA). The primer sequences are shown in Table S1. PCR reactions were performed on a QuantStudio 5 real-time PCR instrument (ThermoFischer Scientific, Waltham, MA, USA) as follows: 95 °C for 3 min, followed by 40 cycles of 95 °C for 5 s, 60 °C for 10 s, and then 1 cycle at 95 °C for 1 min, 55 °C for 5 s, 95 °C for 30 s, and 37 °C for 30 s. In all experiments, β2-microglobulin (*B2M*) was used as the reference housekeeping gene, and results were analyzed using the QuantStudio Design and Analysis software.

### 2.5. Immunofluorescence

The IntraPrep Permeabilization Reagent kit (A07803—Beckman Coulter, Burea, CA, USA) was employed to study the expression of intracellular proteins, whereas antibodies against membrane proteins were used directly. After incubation with primary antibodies against ezrin (#35-7300—Invitrogen), GRHL2 (#MA5-31388—Invitrogen), or RAB25 (#MA5-15587—Invitrogen), a secondary antibody conjugated with Alexa 488 was used. The anti-EpCAM and -CD133 antibodies were directly conjugated with FITC and PE, respectively. Once labeled, cells were deposited on glass slides using a Cytospin 4 centrifuge (Shandon, Runcorn, UK) that were then mounted in ProLong Gold antifade reagent with DAPI (Invitrogen) and analyzed (Axio Imager M1, Carl Zeiss Vision, Halbermoos, Germany). Each antibody has been used and validated independently following the manufacturer’s instructions with the recommended controls.

### 2.6. Bioinformatics and Statistical Analysis

The gene expression data from the colorectal cancer cohort included 390 arrays from primary colon adenocarcinoma, adenoma, metastasis, and the corresponding normal mucosa samples. *GRHL2* mRNA level was obtained from the GEO database (accession number GSE41258). Expression data for normal colorectal epithelium, primary, and metastatic colorectal cancer samples were compared with the Mann–Whitney test.

Statistical significance for the gene expression data was analyzed using one-way analysis of variance (ANOVA). *p* < 0.05 was considered statistically significant. Each experiment was performed at least three times and data are shown as the mean ± SD.

## 3. Results

### 3.1. Expression of SIX1 and Its Co-Activator EYA2 Is Downregulated in All Nine Colon CTC Lines and TGF-β Induction Does Not Alter Their Profiles

**Figure 1:**
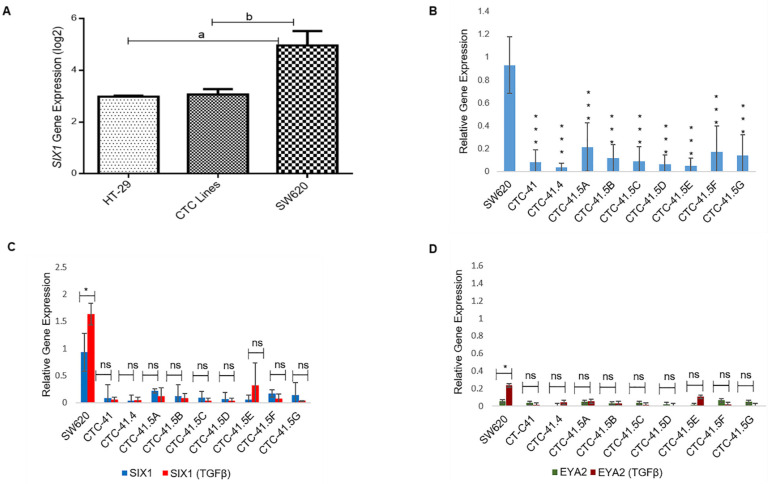
Expression of SIX1 and its co-activator EYA2 in colorectal cancer cell lines in normal culture conditions and after incubation with TGF-β. (**A**) Comparison of SIX1 expression in colon circulating tumor cell (CTC) lines (pooled data), and in primary (HT-29) and metastatic (SW620) colorectal cancer cell lines (microarray data). (**B**) RT-qPCR analysis of SIX1 expression in each of the nine CTC lines and in SW620 cells. (**C**) RT-qPCR analysis of SIX1 expression in cells incubated or not with TGF-β. (**D**) EYA2 expression profile in cells incubated or not with TGF-β. a: *p*-value = 2.73 × 10^−9^; b: *p*-value = 9.80 × 10^−11^. All the RT-qPCR results were normalized to the B2M expression level in each sample; * *p* < 0.05, *** *p* < 0.001, ns: not significan.

The SIX1 transcription factor is important for the mesenchymal profile and drug resistance in various cancer types and is an independent prognostic marker in colorectal cancer [23,24,25]. To investigate SIX1 expression in the nine CTC lines, we compared the microarray expression data obtained in these lines and in the primary HT-29 and metastatic SW620 colon adenocarcinoma cell lines (Figure S2). SIX1 expression was significantly downregulated in HT-29 cells (4.29-fold (log2)) and in all nine CTC lines (3.92-fold (log2)) compared with SW620 cells (Figure 1A). RT-qPCR analysis confirmed that SIX1 expression was significantly lower in the nine CTC lines than in SW620 cells (Figure 1B). SIX1 direct interaction with its co-activator EYA2 gives rise to a transcriptional unit that regulates multiple cancer hallmarks, including cell proliferation, invasion, and resistance to apoptosis [26]. SIX1 and its co-activator EYA2 are often co-overexpressed in tumors and are activated by the TGF-β signaling pathway [26,27]. TGF-β induction is a well-known way to induce EMT in vitro. Therefore, we analyzed SIX1 and EYA2 expression levels after incubation with TGF-β. SIX1 and EYA2 expression levels were increased only in SW620 cells (*p* < 0.05) by incubation with TGF-β, while in the nine CTC lines, no modification was observed for these two EMT markers (Figure 1C,D).

### 3.2. SIX1 Targets Are Expressed in All Nine Colon CTC Lines

**Figure 2:**
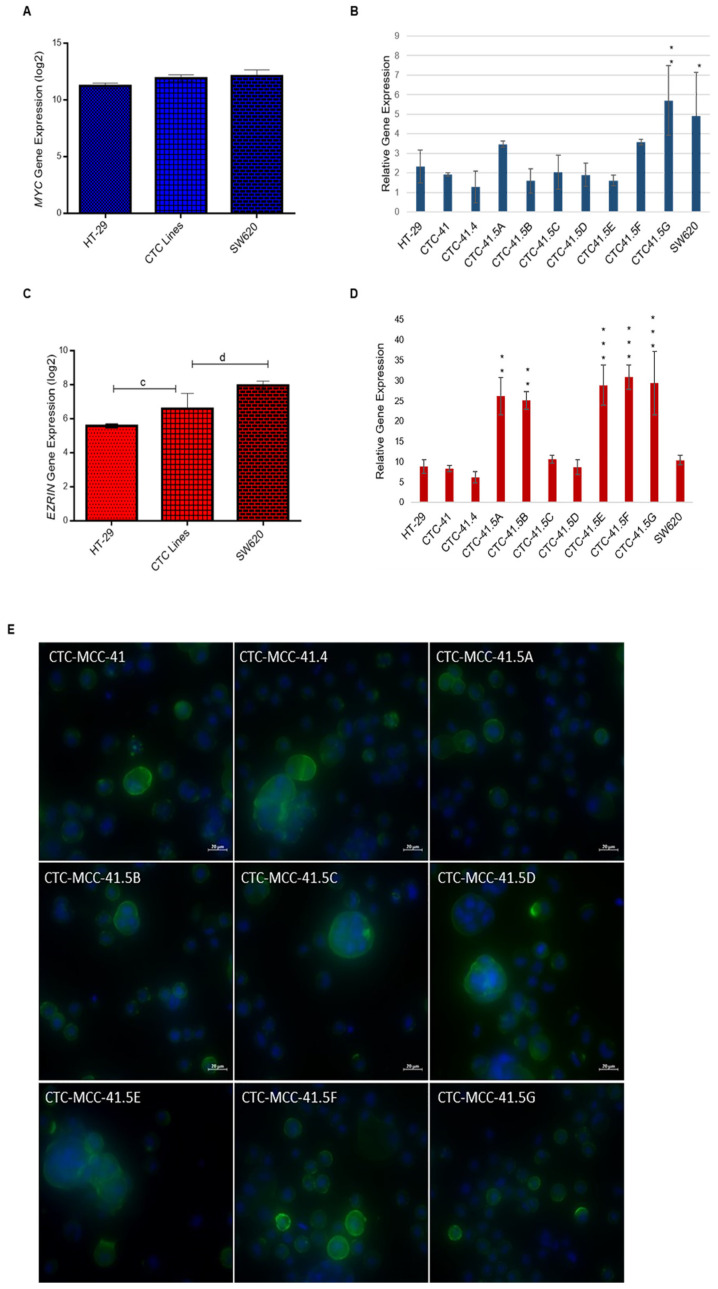
Increased MYC and EZR gene expression in the nine CTC lines compared with HT-29 cells. (**A**) Comparison of MYC expression in CTC lines, HT-29, and SW620 cells (microarray data). (**B**) RT-qPCR analysis of MYC expression in each CTC line and in HT-29 and SW620 cells. (**C**) Comparison of EZR expression in CTC lines, HT-29, and SW620 cells (microarray data). (**D**) EZR expression level in each CTC line, HT-29, and SW620 cells. (**E**) Immunofluorescence analysis of ezrin expression in the nine CTC lines. c: *p*-value = 1.00 × 10^−4^; d: *p*-value = 6.41 × 10^−7^. All RT-qPCR results were normalized to the B2M expression level in each sample; * *p* < 0.05, ** *p* < 0.01, *** *p* < 0.001.

SIX1 activates the transcription of potent oncogenes and stem cell regulators, such as c-MYC and ezrin, that have a vital role in tumor invasion and signal transduction in many cancers [28,29,30,31,32]. MYC has an important role in cell proliferation, differentiation, survival, apoptosis, cancer stemness, and in drug resistance in colorectal cancer stem cells [33]. Comparison of MYC expression in CTCs, HT-29, and SW620 cells showed that it was 1.8-fold (log2) upregulated in CTCs compared with HT-29 cells (Figure 2A). Moreover, all nine CTC lines expressed MYC, but its relative expression was higher in CTC41.5A, CTC41.5F, and particularly in CTC41.5G cells, as well as in SW620 cells, than in the other CTC lines and in HT-29 cells (Figure 2B).

Ezrin, a cytoskeleton regulator protein, is a SIX1 direct transcriptional target in rhabdomyosarcoma [28]. Moreover, SIX1 and ezrin are significant independent prognostic factors in colorectal cancer, and ezrin expression is associated with colorectal cancer metastasis [23,34,35]. Ezrin is implicated in the development, invasion, and metastasis of various human malignancies. A meta-analysis showed that elevated ezrin expression is associated with worse prognosis in patients with different cancer types (e.g., digestive cancers, head and neck squamous cell carcinoma, gynecological cancers, osteosarcoma, hepatobiliary cancer, and non-small cell lung cancer) [36]. EZR (the gene encoding ezrin) was significantly upregulated (2.37-fold (log2)) in all CTC lines compared with HT-29 cells. Conversely, the EZR expression level was 2.23-fold lower in CTCs than in SW620 cells (Figure 2C and Figure S3). RT-qPCR analysis showed similar EZR expression levels in CTC-41, 41.4, 41.5C, and 41.5D cells and in HT-29 cells, whereas they were significantly higher in CTC-41.5A, 41.5B, 41.5E, 41.5F, and 41.5G cells (Figure 2D). We detected ezrin protein in the cytoplasm, particularly near the cell membrane of all CTC cell lines (Figure 2E). Altogether, these data indicate that in our CTC lines, the SIX1 target genes MYC and EZR were upregulated without the need of SIX1 activation.

### 3.3. MET Marker Expression Pattern Analysis Revealed That GRHL2 Is Upregulated in Colon CTC Lines

**Figure 3:**
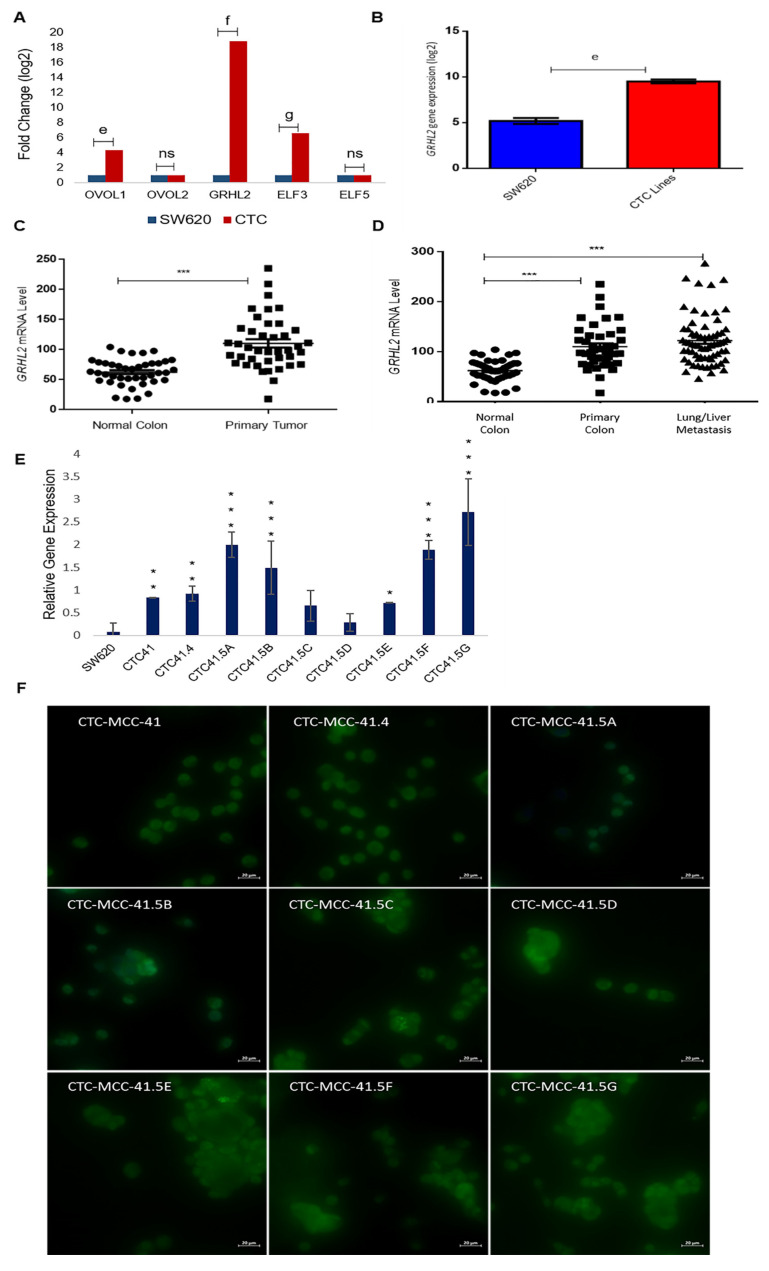
Upregulation of the mesenchymal-to-epithelial transition (MET) marker GRHL2 in the nine colorectal CTC lines and in colorectal cancer samples. (**A**) Comparison of the expression of the indicated MET transcription factors in CTC lines and SW620 cells (microarray data). (**B**) The GRHL2 MET marker is significantly upregulated in CTC lines compared with SW620 cells. (**C**) GRHL2 mRNA level in paired normal colorectal epithelium and primary colorectal cancer samples (*n* = 27). Data are from a cohort of patients with colon neoplasms (GSE41258). (**D**) GRHL2 mRNA level (same cohort as in (**C**)) in normal colorectal epithelium (*n* = 41), primary (*n* = 41), and lung and liver metastatic (*n* = 66) colorectal cancer samples. (**E**) GRHL2 relative gene expression by RT-qPCR in SW620 cells and in each CTC line. (**F**) Immunofluorescence analysis of GRHL2 protein expression in the nine CTC cell lines. e: *p*-value = 2.71 × 10^−9^; f: *p*-value = 1.80 × 10^−17^; g: *p*-value = 1.54 × 10^−12^. All the RT-qPCR results were normalized to the B2M expression level in each sample; * *p* < 0.05, ** *p* < 0.01, *** *p* < 0.001, ns: not significan.

Due to the heterogeneous nature of the observed gene expression patterns in CTCs, we focused on the expression profiles of the GRHL2, ELF3/5, and OVOL1/2, transcription factors involved in MET. Based on the microarray data, GRHL2, ELF3, and OVOL1 were upregulated by 17.8-fold (log2), 5.5-fold (log2), and 3.3-fold (log2) respectively, in CTCs compared with SW620 cells, whereas OVOL2 and ELF5 expression levels were comparable in the two cell types (Figure 3A). We confirmed the strong GRHL2 expression difference between CTC lines and metastatic SW620 cells by RT-qPCR (Figure 3B). The gene array data showed that GRHL2 expression was increased by approximately 4-fold (log2) in all nine CTC lines compared with SW620 cells (Figure S4). Previous in vitro and in vivo studies showed that GRHL2 is suppressed by TGF-β activation [37,38].

Recent studies suggest that a full mesenchymal phenotype is not suitable for metastasis formation, and that GRHL2 may stabilize a hybrid epithelial/mesenchymal phenotype and support cell migration [39,40]. GRHL2 upregulation in the CTC lines prompted us to study its expression in more detail using microarray data (GEO accession number GSE41258) of primary colon adenocarcinoma, metastatic cancer, and the corresponding normal mucosa samples. The GRHL2 mRNA level was significantly increased in colorectal cancer tumor samples compared with the matching normal colorectal samples (Figure 3C). GRHL2 mRNA levels were also significantly increased in primary and lung/liver metastatic CRC samples compared with normal colorectal samples from the same cohort (Figure 3D). Moreover, RT-qPCR analysis showed that GRHL2 expression was upregulated in all nine CTC lines compared with SW620 cells, particularly in CTC-41, CTC-41.4, CTC-41.5A, CTC-41.5B, CTC-41.5E, CTC-41.5F, and CTC-41.5G cells (Figure 3E). Consistent with the mRNA data, we detected GRHL2 protein in the nucleus of all CTC cell lines (Figure 3F). Overall, GRHL2 was upregulated in colorectal cancer and in liver and lung metastatic samples compared with normal colon tissue, and GRHL2 was expressed in all CTC cell lines.

### 3.4. EpCAM and RAB25, Direct Targets of GRHL2, and CD133, Its Indirect Target, Are Differentially Regulated in the Nine Colon CTC Lines

**Figure 4:**
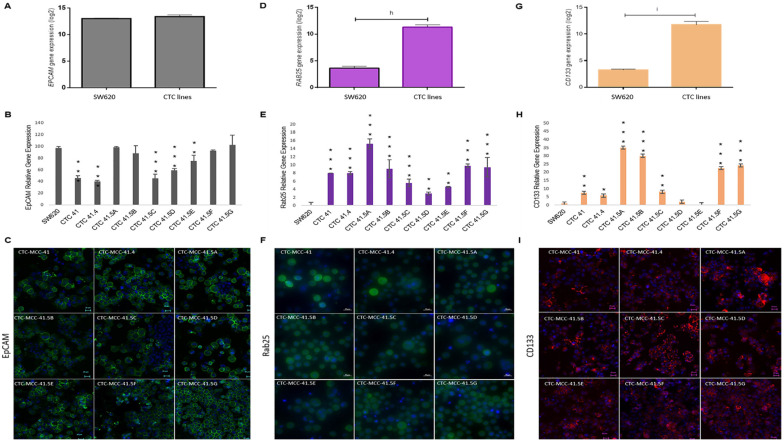
The GRHL2 direct and indirect targets EpCAM, RAB25, and CD133 are differentially expressed in each CTC line. (**A**) Comparison of EPCAM expression in CTC lines and SW620 cells (microarray data). (**B**) Relative EPCAM gene expression in each CTC line and in SW620 cells. (**C**) EpCAM protein expression in the nine CTC lines by immunofluorescence analysis. (**D**) Comparison of RAB25 expression in CTC lines and SW620 cells (microarray data). (**E**) RAB25 gene expression profile in each CTC line and in SW620 cells. (**F**) Immunofluorescence staining of RAB25 protein in the nine CTC cell lines. (**G**) Comparison of CD133 in CTC lines and SW620 cells (microarray data). (**H**) CD133 expression pattern in CTC lines and SW620 cells. (**I**) CD133 protein detection by immunofluorescence analysis. The scale bars denote 20 μm. h: *p*-value = 1.57 × 10^−23^; i: *p*-value = 1.34 × 10^−18^. All the RT-qPCR results were normalized to the B2M expression level in each sample; * *p* < 0.05, ** *p* < 0.01, *** *p* < 0.001 compared with SW620 cells.

A recent study showed that GRHL2 is a key player in EMP [40] and other studies indicated that GRHL2 directly regulates EpCAM and RAB25 expression, which are correlated with cancer progression [41,42,43,44]. Previously, we demonstrated that EpCAM is expressed by all nine colon CTC lines and HT-29 and SW620 cells [19]. We confirmed this result by microarray analysis and found similar expression levels in CTC lines and in metastatic SW620 cells (Figure 4A). However, in the individual CTC lines, EPCAM expression was significantly lower in CTC-41, 41.4, 41.5C, 41.5D, and 41.5E cells compared with SW620 cells (Figure 4B). Immunofluorescence analysis showed that EpCAM protein was strongly expressed by all nine CTC lines (Figure 4C). Moreover, EPCAM and GRHL2 expression profiles were similar in the nine CTC lines. Conversely, SW620 cells showed higher EPCAM expression and lower GRHL2 expression (Figure 3E and Figure 4B).

Another known direct target of GRHL2 is RAB25, a tumor-suppressor gene in colorectal cancer [45]. The RAB25 expression level was significantly higher in CTC lines than in metastatic SW620 cells (Figure 4D). Analysis of RAB25 expression (gene and protein) in the different cell lines (Figure 4E,F and Figure S5) showed no gene expression in SW620 cells, in agreement with a previous study reporting that RAB25 is downregulated by hyper-methylation in this cell line [46]. Conversely, RAB25 was expressed in all nine CTC lines (Figure 4E).

Previous studies reported that GRHL2 directly suppresses the expression of miR-122, a post-transcriptional regulator of CD133 expression [47,48,49]. CD133, a cancer stem cell marker, is a significant prognostic factor of colorectal cancer survival [50]. CD133 was overexpressed in CTC lines compared with SW620 cells (Figure 4G). Moreover, RT-qPCR analysis showed that CD133 expression was high in all CTC lines, except for CTC-41.5D and 41.5E cells (Figure 4H). In addition, immunofluorescence analysis highlighted CD133 protein expression in all CTC lines, similar to EpCAM and RAB25, two direct targets of GRHL2 (Figure 4I).

### 3.5. Downregulation of ZEB1, a Common Target of the MET Markers GRHL2 and ELF3, in the CTC Lines

**Figure 5:**
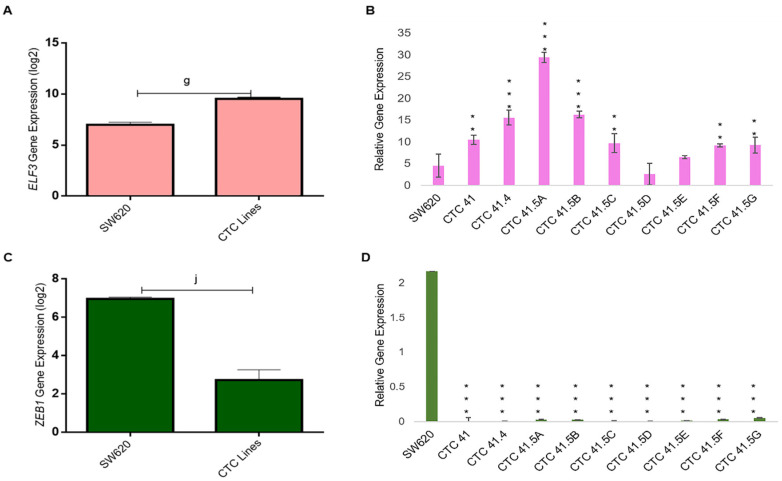
ELF3 and ZEB1 expression. (**A**) ELF3 mRNA level was significantly upregulated in all CTC lines compared with SW620 cells (microarray data). (**B**) RT-qPCR analysis of ELF3 in the nine CTC lines and in SW620 cells. (**C**) Comparison of ZEB1 expression in all CTC lines and in SW620 cells. (**D**) ZEB1 expression analysis by RT-qPCR in the nine CTC lines and in SW620 cells. g: *p*-value = 1.54 × 10^−12^; j: *p*-value = 1.20 × 10^−17^. All the RT-qPCR results were normalized to the B2M expression level in each sample; ** *p* < 0.01, *** *p* < 0.001 compared with SW620 cells.

GRHL2 also regulates the expression of E74-like ETS transcription factor 3 (ELF3), another transcription factor involved in MET [40]. We found that ELF3 was highly expressed in all CTC lines, except in the CTC-41.5D line, and significantly overexpressed compared with SW620 cells, as observed for GRHL2 (Figure 5A,B). GRHL2 and ELF3 have a common target: ZEB1, a well-known EMT inducer. ZEB1 was downregulated (transcriptomic and RT-qPCR analyses) in all nine CTC lines (Figure 5C,D).

## 4. Discussion

EMT is an important cellular program for embryogenesis, wound healing, and malignant progression. In the metastatic process, tumor cells first lose epithelial properties, by undergoing EMT, to leave the tumor, migrate, and intravasate blood vessels to potentially invade a distant organ. Then, tumor cells revert to epithelial characters, through MET, to settle in a secondary distant tissue [51]. Recent evidence indicates that cancer cells have a hybrid mesenchymal and epithelial character and that this EMT/MET plasticity, also called EMP, is associated with their metastatic potential and poor patient outcome [11,52]. CTCs, as cancer cells that circulate through the bloodstream and a source of metastasis precursors, represent the ideal source to study the EMP process. It is of utmost importance to focus our efforts on their identification and characterization to understand the biology of the metastatic cascade [53]. Our group published the first experimental proof of long-term colon CTC lines in 2015 [18]. Our previous work to characterize these nine colon CTC lines, established from the same patient at different time-points during colon cancer progression and treatment, showed that they display an intermediate EMT profile with high plasticity [19]. In the present study, we analyzed EMT and MET markers as well as their target genes in these nine CTC lines, and compared their profiles to those of HT-29 and SW620 cells, two well-known cell lines derived from primary and metastatic colon cancer, respectively.

First, we analyzed the expression of SIX1, an EMT-marker, in the serial CTC lines. This transcription factor is important for the mesenchymal profile and drug resistance in various cancers [23,24,25]. *SIX1* expression was downregulated in our colon CTC lines (RT-qPCR validation of microarray data), even after TGF-β activation, used as an EMT inducer. This result strongly suggests that these tumor cells do not rely on this specific driver during EMT to leave the primary tumor. Similarly, its co-activator *EYA2* was downregulated in the nine CTC lines in basal conditions and after incubation with TGF-β. Conversely, *MYC* and *EZR*, SIX1 oncogenic target genes, were upregulated without the need of SIX1 activation. MYC has an important role in cell proliferation, differentiation, survival, apoptosis, cancer stemness, as well as in drug resistance in colorectal cancer stem cells [33]. Moreover, ezrin promotes invasion and metastasis of cancer cells [54]. A previous study showed that in different cancer types, deregulation of gene expression could involve various transcriptional and/or post-transcriptional regulation mechanisms [55]. These results imply that even in the absence of well-known and described regulators (i.e., SIX1), tumor cells may still develop alternative processes to activate genes that promote their plasticity and invasiveness.

EMT is a key step to increase the motility and the invasiveness of cancer cell subsets in the primary tumor; however, for the crucial last step of metastatic colonization in a distant organ, cancer cells need to revert to an epithelial phenotype [56,57]. The reverse process of EMT, known as MET, is orchestrated by MET-transcription factors, such as OVOL1/2, GRHL2, ELF3, and ELF5 [14]. Here, we found that *GRHL2*, *ELF3*, and *OVOL1* were overexpressed in the CTC lines compared with SW620 cells (metastatic colorectal cancer cell line), particularly *GRHL2*. *GRHL2* was also upregulated in colorectal cancer and in liver and lung metastatic samples compared with normal colon tissue, and GRHL2 was expressed in all CTC cell lines. Recent papers indicate that a full mesenchymal phenotype is insufficient for metastasis formation, and that GRHL2 may stabilize the hybrid epithelial/mesenchymal phenotype and support cell migration [39,40]. As a transcription factor, GRHL2 is implicated in the regulation of different cellular processes during development, such as epithelial morphogenesis, barrier formation, and wound healing [58]. Therefore, we investigated the expression of GRHL2 target genes that have roles in cancer progression. We found that *RAB25* expression was significantly increased in all CTC lines compared with SW620 cells. Concordantly, a previous study reported that RAB25 is downregulated by hyper-methylation in this cell line [46]. RAB25 is involved in the regulation of epithelial morphogenesis through the control of CLDN4 [59], and its overexpression has been linked to metastatic potential, anchorage-independent cell proliferation, apoptosis, and anoikis prevention, all crucial functions for CTC survival and metastatic competence [60,61]. However, RAB25’s role in cancer remains unclear. Indeed, in various cancer types (e.g., bladder, non-small cell lung, ovarian, prostate cancer, and clear cell renal cell carcinoma) [62,63], RAB25 overexpression has been associated with shorter overall survival. Conversely, in colorectal cancer, RAB25 expression is decreased, and this loss has been associated with poorer survival [46,64]. A recent study introduced a new perspective on the role of small GTPases in EMP, and RAB11 (an evolutionarily conserved protein that belongs to the same family as RAB25) has an important role in the re-localization of epithelial proteins during EMP and also in promoting CTC cluster formation [65].

We observed that CD133 was expressed in CTC lines and its expression profile was positively correlated with that of GRHL2. Although no study showed that GRHL2 directly regulates CD133, in our CTC lines, the CD133 expression profile was similar to that of GRHL2. This may be explained by an indirect regulation through microRNAs, such as miR-122 [47,48]. CD133 is a cancer stem cell marker, and CD133-expressing cells exhibit self-renewal potential and the ability to regenerate a histologically similar tumor mass following transplantation into immunodeficient mice. Moreover, CD133 expression in cancer cells has been correlated with poor prognosis and reduced overall survival in many different cancers, including colorectal cancer [66,67,68]. We hypothesize that GRHL2, a MET inducer, activates CD133 to promote CTC stemness and to allow CTCs to settle and grow in a new distant organ where they will form a secondary tumor.

Another MET transcription factor, ELF3, was overexpressed in our nine CTC lines compared with the metastatic cell line SW620. This factor has been identified as a GRHL2 target. ELF3 is overexpressed in colorectal cancer and promotes colorectal cancer progression by transactivation of β-catenin [41,69].

Finally, the MET transcription factors ELF3 and GRHL2 are both negative regulators of ZEB1.This is consistent with ELF3 and GRHL2 overexpression, but also with the lower SIX1 and EYA2 expression levels in CTCs than in SW620 cells, implying that these cells do not display a full mesenchymal phenotype.

**Figure 6:**
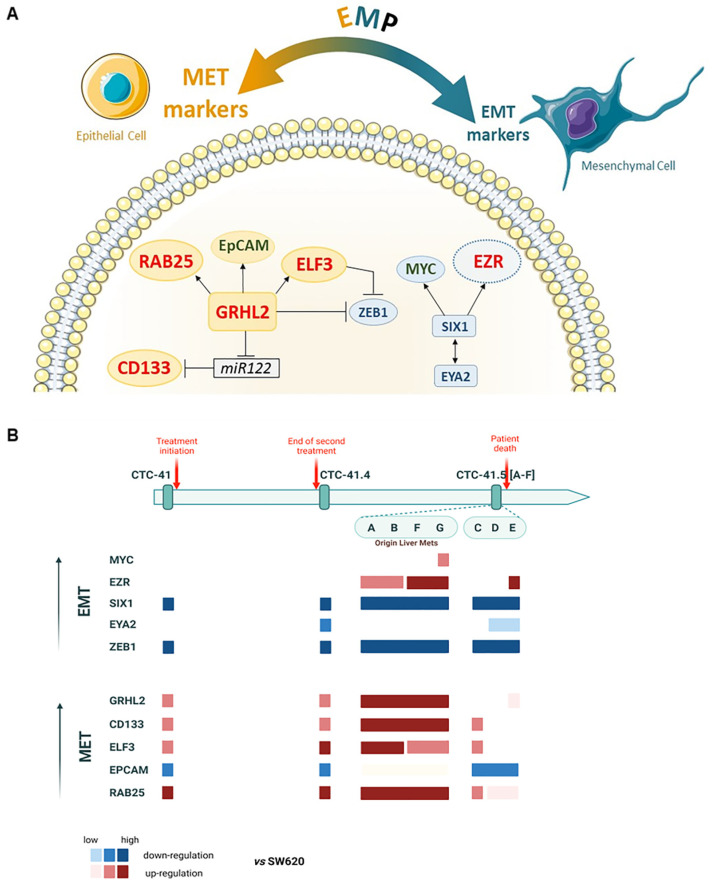
Schematic presentation of EMP marker expression in the nine serial CTC lines. (**A**) Network and regulations; in red, significantly upregulated genes (>2-fold change in log2); in green, upregulated genes but without significant difference; in blue, genes significantly downregulated in the nine CTC lines compared with the metastatic colorectal cancer SW620 cell line. (**B**) Upregulation of EMT (in blue) and MET (in red) markers in each CTC line, compared to SW620, showing the EMP profile during disease progression and treatment.

Altogether, these results suggest that our colon CTC lines have acquired some mesenchymal features to migrate and intravasate, but they remain more in an epithelial state, as indicated by the overexpression of MET inducers, to be able to colonize distant organs when required. Indeed, overall, EMT inducers were downregulated, whereas MET markers were upregulated, although some EMT-inducer targets were still overexpressed (Figure 6A). Specifically, these MET markers were more expressed in the subgroup CTC-MCC-41.5 (A, B, F, G) (Figure 6B) that originate from liver metastases [70]. Therefore, these results indicate that this subgroup has already undergone MET.

## 5. Conclusions

This study improved our knowledge on the heterogeneity and the plasticity of these special subsets of CTC clones selected during cancer progression and treatment pressure. These findings should help to better understand CTC clonal evolution over time in a patient with metastatic colon cancer who was treated with different therapies and who relapsed several times until death. Advances in the knowledge of the plasticity of these metastasis-initiator cells will provide new ideas for the development of innovative personalized treatments for colorectal cancer.

## Data Availability

The datasets used and analyzed during the current study are available from the corresponding author upon reasonable request.

## Supplementary Materials

The following are available online at https://www.mdpi.com/article/10.3390/cancers13215408/s1, Figure S1: Timeline of sequential CTC line derivation from a colorectal cancer patient. Figure S2: GeneChip assay of SIX1 mRNA level in HT-29, SW620, and CTC cell clones. Figure S3: EZRIN gene expression profile by GeneChIP assay. Figure S4: GeneChip assay of GRHL2 mRNA level in CTC-MCC lines and SW620 cells. Figure S5: RAB25 gene expression profile by GeneChIP assay. Table S1: The sequence of the forward and reverse primers used for RT-qPCR analysis.
