# Supplementary material for: Epithelial-to-Mesenchymal Plasticity in Circulating Tumor Cell Lines Sequentially Derived from a Patient with Colorectal Cancer
Source: Cancers (Basel). 2021 Oct 28;13(21):5408. doi: 10.3390/cancers13215408 (PMC8582537; doi:10.3390/cancers13215408)
Supplement: Supplementary file 1 [file cancers-13-05408-s001.zip › cancers-1339958-supplementary/Table S1.pdf]

Table S1. The sequence of the forward and reverse primers used for qRT-PCR analysis.

Gene		Sequence 5' to 3'	
SIX1	Forward Sequence	AAAGGGAAGGAGAACAAGGATAG	NM_005982.4
	Reverse Sequence	AGCCTACATGATTACTGGGATTT	
EYA2	Forward Sequence	CAGAGTGGATTCCTCAGCTATG	NM_005244.5
	Reverse Sequence	CCTCCTTGATAGAACCCTGTTG	
c-*MYC*	Forward Sequence	GCTGCTTAGACGCTGGATTT	NM_002467.4
	Reverse Sequence	GAGTCGTAGTCGAGGTCATAGTT	
EZRIN	Forward Sequence	CCGTGGGATGCTCAAAGATAA	NM_003379.5
	Reverse Sequence	TCCAAGCCAAAGGTCTGTTC	
GRHL2	Forward Sequence	CGTCTGGGTGCAGTAGTTATAG	NM_024915.4
	Reverse Sequence	GGGAACCCTGGAGGTATTTAG	
EPCAM	Forward Sequence	ACAAGGACACTGAAATAACCTGC	NM_002354
	Reverse Sequence	TTTTGAGAAGAATTTTGAACCAGAT	
RAB25	Forward Sequence	GGGAATGGAACTGAGGAAGATTA	NM_020387.4

	Reverse Sequence	CGTGAATCGGGAGAGTAGATTG	
CD133	Forward Sequence	TTGAATGAAACTCCAGAGCAAA	NM_001145847
	Reverse Sequence	CCTCCTAGCACTGAATTGATACTG	
ELF3	Forward Sequence	TCAACGAGGGCCTCATGAA	NM_004433.5
	Reverse Sequence	TCGGAGCGCAGGAACTTG	
ZEB1	Forward Sequence	TGCACTGAGTGTGGAAAAGC	NM_001128128.3
	Reverse Sequence	TGGTGATGCTGAAAGAGACG	
B2M	Forward Sequence	GTCTTTCAGCAAGGACTGGTCT	NM_004048
	Reverse	TTACATGTCTCGATCCCACTTAAC	
