# Supplementary figures and images for: Epithelial-to-Mesenchymal Plasticity in Circulating Tumor Cell Lines Sequentially Derived from a Patient with Colorectal Cancer
Source: Cancers (Basel). 2021 Oct 28;13(21):5408. doi: 10.3390/cancers13215408 (PMC8582537; doi:10.3390/cancers13215408)

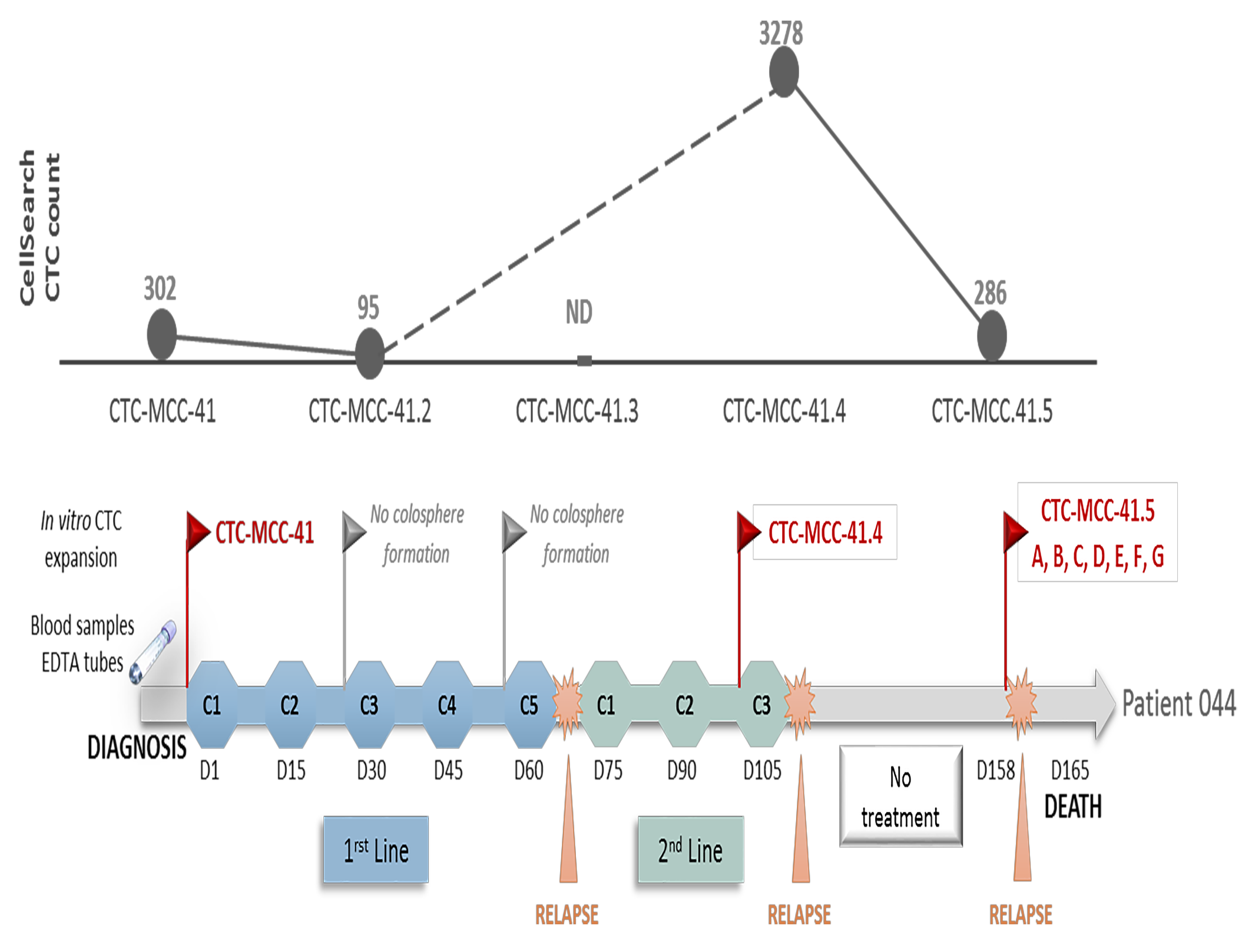

Supplement: Supplementary file 1 [file cancers-13-05408-s001.zip › cancers-1339958-supplementary/Figure S1.tif]
